# Effect of Intracochlear Brain-Derived Neurotrophic Factor on Guinea Pig Sensorineural Hearing Loss

**DOI:** 10.1177/19160216251336679

**Published:** 2025-06-30

**Authors:** Deanna Gigliotti, Brian Blakley

**Affiliations:** 1 Department of Otolaryngology—Head & Neck Surgery, University of Manitoba, Winnipeg, MB, Canada

**Keywords:** BDNF, sensorineural hearing loss, otology/neurotology, intracochlear, neurotrophic factor

## Abstract

**Importance:**

This study investigates the potential of brain-derived neurotrophic factor (BDNF) as treatment for sensorineural hearing loss (SNHL) in a guinea pig model to potentially advance hearing restoration strategies. The correlation between oxidation-reduction (REDOX) potential in blood and perilymph is evaluated to confirm using blood as a proxy for perilymph in further study.

**Objectives:**

To evaluate hearing following 2 intracochlear applications of BDNF as a therapy for hearing loss. To evaluate for correlation in REDOX potential of perilymph and auditory brainstem response (ABR).

**Study Design:**

Positive-control animal preclinical study.

**Setting:**

Translational laboratory science.

**Participants:**

Animal model (guinea pigs).

**Intervention or Exposures:**

SNHL was created in 15 guinea pigs using intraperitoneal cisplatin (CDDP). SNHL was confirmed via ABR testing. Left ears received 2 intracochlear applications of BDNF in varying doses, 30 days apart. Right ears received saline as controls.

**Main Outcome Measures:**

Hearing threshold was determined using ABR testing. Animals underwent terminal surgery to measure the REDOX potential in cerebrospinal fluid (CSF) and blood. Analysis of variance for repeated measures using the SPSS v27 software was employed.

**Results:**

Variable, subtotal hearing loss was established utilizing CDDP. Animal ABR thresholds after CDDP, prior to first BDNF application, were worse than baseline. There was no improvement in hearing thresholds when treated and nontreated ears were compared. Varying doses of BDNF did not produce differences in hearing thresholds. The REDOX potential of perilymph, blood, and CSF correlate in the same animal; however, the values themselves were significantly different.

**Conclusions and Relevance:**

There is no improvement in guinea pig hearing with 2 intracochlear applications of BDNF when applied as described in this paper. Previous work suggested possible subclinical gain with 1 application; however, with 2 applications we found no improvement. The REDOX potential of blood and CSF correlates within an animal, suggesting blood may be used as a proxy for REDOX measures in perilymph.

## Graphical abstract

**Graphical Abstract:**
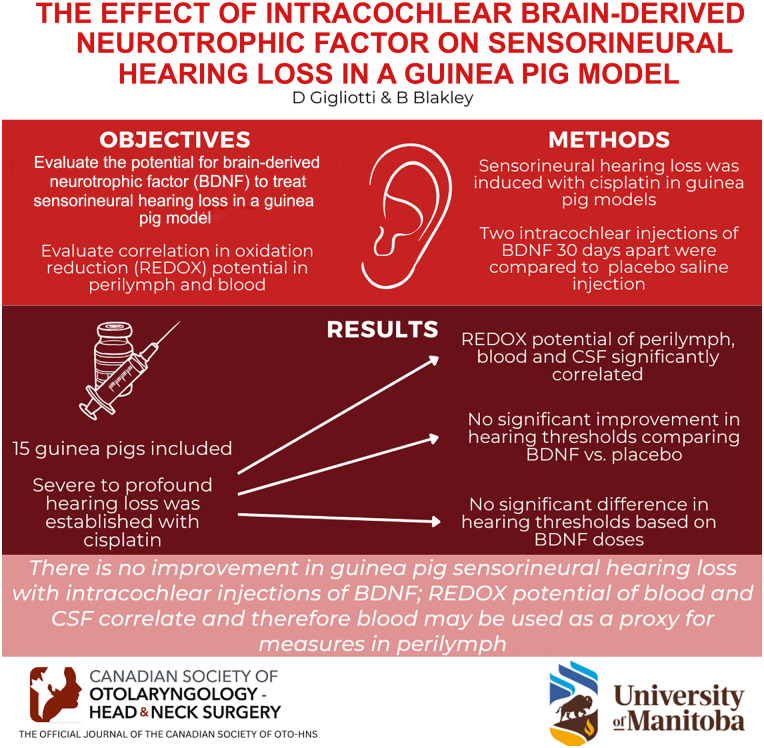

## Introduction

The neurotrophic hypothesis suggests a deficit of neurotrophins accounts for all age-related degeneration in the central nervous system.^1^ Neurotrophins, including brain-derived neurotrophic factor (BDNF), enhance cell growth of neurons, and BDNF has been shown to support structural regeneration of mammalian hair cells.^2^ This research project explores neurotrophic-based strategies for the treatment of sensorineural hearing loss (SNHL) in animals using a model that could be applied to humans. Single intracochlear applications of BDNF have resulted in a subclinical gain of hearing in the setting of SNHL.^3,4^ The purpose of this study was to build on that previous research. It also sought to support or refute our previous laboratory finding that the oxidation-reduction (REDOX) potential of blood and CSF are correlated within an animal. Confirmation of correlation would suggest that blood measurements of REDOX potential could be substituted for perilymph measurement, which would simplify the study of REDOX potentials in human ears.

SNHL is one of the most common disabilities in society and is associated with loss of quality of life and loss of economic productivity.^5^ As the population ages and the number of elderly increases, hearing loss will become much more frequent. A survey in 2004 reported that the incidence of hearing loss in Americans was 16.1% with males affected 5.5 times more often than females.^6^ About 10% of the population has a significant hearing loss, and the number of Americans with hearing loss has doubled in the past 30 years.^7,8^ It is already a serious public health problem associated with great loss of quality of life, social problems, lost employment, and significant loss of economic productivity that will increase markedly in the years to come.^9^

Direct application of BDNF to the round window after puncture was employed because the molecular weight of BDNF (27.5 kDa) is such that it would be unlikely to cross the blood-labyrinth barrier if administered systemically. Instead, the drug was introduced directed into the inner ear, with the injection site plugged after each application. As in previous work, we implemented a “soft technique” whereby the drug was introduced to the cochlea after slow, gentle, shallow puncture of the round window.

The impact of this knowledge will improve our understanding of the effect of intracochlear administration of neurotrophins such as BDNF on SNHL.

While human application is desired, we still must quantify promising strategies in animals before proceeding to human clinical trials. This project extends on research for which the principal investigator has already developed models and methods.

## Methods

A positive-control experimental animal study was undertaken after appropriate ethics approval from Animal Ethics Board. Stable, severe SNHL was created in 15 guinea pigs using intraperitoneal cisplatin (CDDP; 4 mg/kg × 3 doses on alternate days—total dose 12 mg/kg). In previous studies, this dose has been associated with acceptable mortality if supplemental fluids are carefully administered for the first day after injection. Sample size of 15 animals was utilized after power analysis suggested 12 animals/group would detect a 15 dB change in hearing with 80% power. We assumed a maximum data loss rate of 25%. Creation of SNHL was confirmed via auditory brainstem response (ABR) threshold testing in a sound booth, at 3, 6, 12, and 24 K under ketamine anesthesia. The ABR protocol is previously described.^3^ The left ears received 2 intracochlear applications of BDNF (Sigma Corp (St. Louis, MO, USA); ranging from 0.05 to 2.0 µg in 0.01 cc), 30 days apart. The right ears received 2 applications of equal volumes of saline to function as controls. The protocol for injections is previously described.^3^ It utilized BDNF and saline inserted through the round window after the removal of the tympanic membrane to provide consistent, unavoidable conductive hearing loss in all animals.

After the application of BDNF, ABR threshold testing was repeated at the 30th day (prior to the first application of BDNF), at the 60th day (prior to the second application of BDNF), and finally at the 90th day. Study timeline is articulated in Supplemental Figure 1.

Under terminal anesthetic CSF was obtained by piercing the dura of the foramen after suboccipital dissection, and cardiac puncture was used to obtain blood for REDOX measures or CSF and blood. All REDOX potentials were measured with the REDOX probe by Unisense (Aarhus, Denmark). Perilymph REDOX potentials were measured by Unisense with the reference electrode in the flank and the sensor traversing the round window.

Changes in auditory threshold at baseline compared with post-hearing loss and post-BDNF treatment was assessed by the analysis of variance for repeated measures using the SPSSv27 software (IBM Corp., Armonk, NY).

Analysis of variance for repeated measures was carried out using ABR threshold as the dependent variable and frequency (3, 6, 12, and 24 kHz), ear (BDNF or saline) as within-subject variables and BDNF dose (0, 0.05, 0.5, 1.0, and 2.0 µg) as a between-subject variable, across the 4 time periods (baseline, after CDDP, 30 days after first BDNF treatment, and 30 days after the second BDNF treatment). Mauchly’s test of sphericity was not significant, indicating that a parametric analysis is valid. The correlation between REDOX potentials was evaluated using Pearson’s correlation coefficient.

## Results

**Figure 1.**
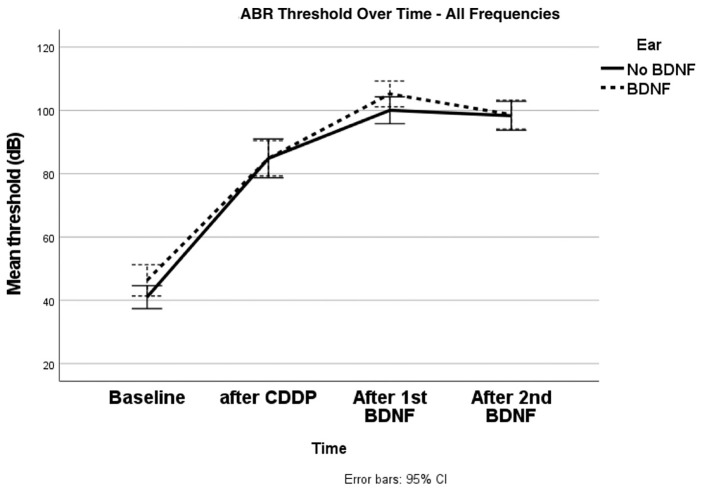
Mean hearing threshold (dB) at each of the tested time intervals; baseline, at the 30th day after the stabilization of hearing loss post-CDDP, at the 60th day—equivalent to 30 days post-first BDNF, and finally at the 90th day, after the second application of BDNF. Control (saline) ears are shown as solid line (“no BDNF”) and BDNF ear is shown in dotted-line. BDNF, brain-derived neurotrophic factor; CDDP, cisplatin.

Severe to profound hearing loss was established utilizing intraperitoneal CDDP. Mean ABR thresholds after CDDP, prior to first BDNF application were worse than baseline thresholds. There was no difference in ABR thresholds in ears treated with intracochlear application of BDNF compared with saline application (Figure 1). Furthermore, varying doses of BDNF did not produce differences in hearing thresholds (*P* = .51).

The REDOX potential of blood and CSF correlates significantly in the same animal. There was a positive correlation between variables, *r*(118) = .30, *P* = .011, although REDOX potentials were significantly different, *P* = .001. The final hearing threshold does not correlate with either REDOX potential, *P* = .279 and .878, for threshold versus CSF and blood, respectively.

MANOVA for repeated measures was conducted assigning “ABR threshold” (dB) as the dependent variable and 4 independent variables: 1 between-subject factor and 3 within-subject factors. The between subjects factors was “BDNF dose” (0.05, 0.5, 1.0, or 2.0 ug). The 3 within subjects factors were “time” (baseline, after CDDP, after first BDNF, after second BDNF), “ear” (BDNF or saline treated) and “frequency” (3, 6, 12, and 24 kHz).

Threshold changes over time were significant (*P* = .001) as expected and across frequencies (*P* = .02), but not between the factors of most interest in this study: “ear” (*P* = .291) and “BDNF dose” (*P* = .784) or the interaction between “ear” by “BDNF dose” (*P* = .510).

## Discussion

The findings of this study shed light on several key aspects of neurotrophic factor therapy for hearing loss induced by ototoxic insults, specifically focusing on the role of BDNF and its potential efficacy in mitigating cochlear damage induced by CDDP. Our investigation revealed intriguing insights into the relationship between blood and CSF REDOX potentials, and the lack of effect BDNF treatment in varying dosages.

Firstly, our investigation into the efficacy of BDNF treatment revealed interesting results regarding its effectiveness in mitigating CDDP-induced hearing loss. While we did not observe significant differences in hearing threshold with varying doses of BDNF, our data suggest that the severity of cochlear insult induced by CDDP may influence the therapeutic response to BDNF. The relatively-high threshold change (~45 dB) induced by CDDP in our study may indicate that the extent of cochlear damage exceeds the therapeutic capacity of BDNF within the tested dosage range, if such therapeutic capacity exists. Future studies exploring the dose-response relationship of BDNF in varying degrees of cochlear insult are warranted to elucidate optimal treatment parameters.

Previous studies have reported age-related loss of BDNF expression in the cochlea, highlighting the importance of considering age-related factors in the development of therapeutic interventions for hearing loss.^10^ The gradual decline in BDNF expression during the later stages of life underscores the need for early-intervention strategies to preserve auditory function and mitigate age-related cochlear degeneration.

Moreover, our findings provide further information regarding minimizing middle ear trauma during drug application. The destruction of the tympanic membrane in our experimental model may have compromised the integrity of the middle ear. In a study reviewing the effect of tympanic membrane perforation with BDNF delivery, perforation did not lead to collateral cochlear damage.^11^ The current data provide valuable insights into the safety profile of invasive cochlear drug delivery methods. Future research directions can review the effect of tympanic membrane condition and efficacy. Thereafter, minimally-invasive techniques for drug administration may or may not be required to mitigate potential iatrogenic damage and enhance treatment outcomes.

Our observation of a correlation between blood and CSF biomarkers supports the notion that blood may serve as a viable proxy for monitoring biochemical changes in CSF. This finding underscores the potential utility of blood-based biomarkers in assessing central nervous system conditions, including those affecting auditory function. This finding is supported with those reported in the literature.^4^ This observation suggests that future studies could involve consideration of assessing the REDOX potential of blood when evaluating possible therapies for hearing loss without violating the tympanic membrane to assess perilymph.

However, despite potential correlations, our data also indicate distinct compositional differences between CSF and blood, as evidenced by variations in REDOX potentials. This highlights the importance of considering the unique biochemical milieu of CSF when developing therapeutic strategies targeting the central auditory pathway. Understanding the implications of REDOX physiology in different tissues may prove to be clinically beneficial.

## Conclusion

Our study contributes to the growing body of literature on neurotrophic factor therapy for hearing loss and provides valuable insights into the complexities of cochlear neuroprotection and the challenges associated with translating preclinical findings into clinical practice. As employed in this study intracochlear administration of BDNF does not appear to be a viable treatment option for established SNHL. Differences in the REDOX potential among various physiological compartments exist and may be valuable for future research.

## Supplemental Material

sj-pdf-1-ohn-10.1177_19160216251336679 – Supplemental material for Effect of Intracochlear Brain-Derived Neurotrophic Factor on Guinea Pig Sensorineural Hearing Loss Supplemental material, sj-pdf-1-ohn-10.1177_19160216251336679 for Effect of Intracochlear Brain-Derived Neurotrophic Factor on Guinea Pig Sensorineural Hearing Loss by Deanna Gigliotti and Brian Blakley in Journal of Otolaryngology - Head & Neck Surgery
