# Supplementary material for: Effect of Intracochlear Brain-Derived Neurotrophic Factor on Guinea Pig Sensorineural Hearing Loss
Source: J Otolaryngol Head Neck Surg. 2025 Jun 30;54:19160216251336679. doi: 10.1177/19160216251336679 (PMC12209572; doi:10.1177/19160216251336679)
Supplement: sj-pdf-1-ohn-10.1177_19160216251336679 – Supplemental material for Effect of Intracochlear Brain-Derived Neurotrophic Factor on Guinea Pig Sensorineural Hearing Loss [file sj-pdf-1-ohn-10.1177_19160216251336679.pdf]

Day 0	Days 3-9	Day 39	Day 69	Day 99
Acquisition Acclimatization	CDDP x3 doses, alternate days	ABR then first BDNF/saline	ABR then second BDNF/saline	Final ABR REDOX measurements
